# Cerebral Syphilis

**Published:** 1887-02

**Authors:** 


### Editorial.

### CEREBRAL SYPHILIS.

That syphilis ever attacks the nervous system seems to have been entirely overlooked by the early writers, and when it was at last discovered "that various nervous complaints, such as paralysis, epilepsy and insanity frequently occurred in those who had previously suffered from the more ordinary forms of constitutional syphilis, this was explained by the convenient figment, a metastasis having taken place from the skin and mucous membrane." This, doubtless, was the notion entertained by Hunter, Astley Cooper and others, who "stated plainly that the brain, as well as the other internal organs, were unsusceptible to the venereal poison," for they could not have denied the fact that syphilitics had peculiar cerebral manifestations. Again, there were those who would explain the peculiar symptoms of the convulsive form of this disease by the vitiated blood sent to the brain, as in uræmia, rather than by any organic change. That syphilis does attack the brain, spinal cord and nerves, is now well established. Whether it was of so frequent occurrence in early years, and not recognized, or whether the disease did not exist, it is difficult to state. Certain it is that this nervous system of our times is more susceptible to disease than formerly, and entirely new forms of troubles are seen. This might explain the so frequent occurrence of the disease at the present time. The literature on this subject is extremely meagre, considering all that has been written on the malady itself. Althaus says that during his studies among the universities of the Continent in 1853 to 1855, he never heard any reference to syphilis of the brain by such masters as Romberg, Von Baerensprung, Sigmund, Skoda and Oppolzer. He says: "The only man who, about that time, knew that the brain was liable to specific disease, was Professor Waller, of Prague, whose lectures I attended in the summer of 1856. He told his hearers then that he had seen red and white softening, and a deposit of solid effusions in the white as well as the grey matter of the brain. According to him, the symptoms during life were indistinct, and we could only diagnose cerebral syphilis where the patient suffered at the same time from external manifestations of the distemper. He had known patients in the later stages of the disease to suffer from epilepsy, paralysis and mental hebetude; but the brain might also suffer, at an early period, sychronously with the ulcerated throat, and he had seen cases in which a most violent form of headache was the principal symptom, and where the patient, in spite of active treatment, had died comatose. I believe that these observations of Waller have never been published, and what I have just mentioned is culled from notes which I took at the time his lectures were delivered." As to the impossibility of diagnosis without external manifestations, this may hold good even at the present time in some forms, while in others the diagnosis is positive without any such assistance, many cases appearing as the only secondary or tertiary manifestations the patient has ever had. In 1860, we had a number of independent and excellent treatises on this subject by Lagneau, L. Gros and Lancereaux, Lambaco and others. They were defective in that the various forms were confused, and as the pathology was only made clear at a later date, it was necessarily defective. Along with the pathological researches of Virchow in 1869, and Heubner in 1874, came a better understanding of the disease. "These researches may, each in its own way, be considered as landmarks in the history of cerebral syphilis. The foundations of the doctrine were now securely laid, so as to be beyond cavil; and it became clear that, although subsequent work might amplify our knowledge in this respect, it could no longer reverse what was once firmly established. The features of this work is, that the peculiar and specific alteration which occurs in the brain as a consequence of constitutional syphilis is not inflammation, but that it bears throughout the character of a neoplasm. This neoplasm may be deposited in any membrane of the brain, forming a tumor or gumma, or it may be so deposited around an artery as to act as a thrombus." It may be deposited in the brain tissue itself, but that encephalitis may follow is a mooted question. Althaus says the great majority of cases are either gummatous or thrombotic in character. He does not call attention to the fact that both of these forms may exist in the same case, each causing a different chain of symptoms, which, unless duly considered, will cause considerable trouble in our diagnosis. The writer, who has seen twelve patients suffering with this disease within an incredibly short time, had one in which both of these pathological conditions obtained in the same patient. At times, while under treatment, the one set of symptoms seem to predominate, and again the other would gain the mastery. In 1879, Forunier published a most admirable treatise on this subject, and since this time, much has been said concerning this disease, especially in the French journals. The claim was made by him, and re-asserted by Althaus in a recent article, that fully eighty per cent. of brain diseases occurring in all patients between the ages of twenty-five and forty, are actually owing to this distemper. While this is exceedingly large, we must admit that this is the cause of an alarming number of brain diseases. " Forunier has distinguished six different forms of it, viz.: the cephalalgic, the congestive, the convulsive or epileptiform, aphasic, the mental, the paralytic; and his description of the symptoms which occur in these several forms is more minute and exhaustive than any other which has been given before, and will, on that account always remain a monument of able, painstaking industry. A disappointing feature of Forunier's work is that it does not generally point out clearly the connection which exists between clinical signs and pathological lesions, and appears to be only slightly acquainted with the modern doctrine of cerebral localization, which just in this department of clinical medicine finds its most interesting and striking practical applications." Dr. Althaus has given us one of the finest treatises on the subject of syphilitic coma and syphilitic hemeplegia, published in the *Medical Record* in October. It can be truly said that syphilitic coma was little understood before. So far as my observation goes, the convulsive or epileptiform is the most frequent and next the cephalalgic, the paralytic, the aphasic, the mental and coma occurring in frequency in the order mentioned. The cephalalgic variety is frequently a prodomatic of the other forms, and may precede them many weeks, or even months. This pain comes on at night, is localized, and has several characteristics, "according to a judicious observer:

1. A severe pain, with a sensation of weight.

2. Constrictive pain, seeming to the patient as though the head were about to split open.

3. Pains, as if from blows with a hammer; instantaneous, and extremely severe."

This form often disappears of itself, only to return in the same manner as at first, and with increased severity.

Syphilitic coma, as has been said, is rare, and as it is little understood at the present time, we give Dr. Althaus on this subject entire:

"That coma, coming on more or less suddenly in an apparently healthy man, or in one who shows at the time unmistakable symptoms of venereal disease, may be a manifestation of this latter distemper, is not generally known in the profession. We are all familiar with uræmic, alcoholic, and diabetic coma: with the coma of cerebral hemorrhage and that of opium-poisoning; with that which occurs after the epileptic fit; with or after severe hysterical, and hystero-epileptic convulsions; after prolonged exposure to extremes of temperature, whether heat or cold; after erysipelas of the face and head from compression of the brain by a depressed fracture of the skull, by extravasated blood, meningitis, and the presence of pus and other products of inflammation. Nor must we forget that several chronic affections of the nervous system, more especially tabes spinalis, and general paralysis of the insane, are apt, toward their termination, to be attended by attacks of coma. Most of these conditions are described in the text-books, while syphilitic coma is not even mentioned. Forunier is almost the only author who has pointedly, although shortly, alluded to this condition, and related an example of it which occurred in his practice. A knowledge of syphilitic coma is, however, of great practical importance, inasmuch as it requires an entirely different treatment from that of other forms of coma, and an incorrect diagnosis is in such a case likely to seal the fate of the patient.

"I have seen, altogether, eight unmistakable cases of syphilitic coma. They all occurred in males between twenty-five and forty-two years of age. In every one of them was there a definite history of primary and secondary syphilis ; in four there was at the time a specific rash on the scalp and other portions of the skin ; and in one an ulcer in the tongue. In one case the coma appeared eight months after infection ; in six between three and five years, and in one case seventeen years afterward. In two cases no other cerebral symptoms had occurred before the coma, while six other patients had at various times suffered from giddiness, epileptiform convulsions, and transient loss of power in the limbs.

"Among the exciting causes of the attack I have noticed overwork, anxiety, trouble, and sexual and alcoholic excesses. In two cases no exciting cause whatever could be ascertained. Six of the patients were professional men, and two were men without any regular occupation.

"The symptoms of syphilitic coma I venture to classify as, first, premonitory signs ; second, symptoms of the initial stage ; and third, symptoms of the final stage of coma.

"1. I have noted the following premonitory symptoms of the attack of coma ; headache, a feeling of confusion and drowsiness, indistinct utterance, a perception of black specks floating before the eyes, with sudden loss of sight for a short time, numbness in the limbs, and some loss of muscular power. In six cases such symptoms occurred either a few hours, or a day or two, before the attack, while in two other cases they appeared to have been entirely absent.

"2. The initial stage of syphilitic coma appears to set in habitually during sleep, the patient being discovered by his friends or servants in the morning in a state of apparent insensibility, from which he cannot be roused. He is lying quietly on his back, apparently quite unconscious, and, as it were, in a profound sleep. He is evidently not suffering any pain; he does not moan, throw himself about, or put his hands to his head. The face is absolutely devoid of expression; there is a complete blank, and no distortion of the features. The complexion is generally pale. Sometimes he can be roused by shouting to him; he may speak a word or two, and appears to recognize the voice of a friend better than that of a stranger. When asked whether he can see you, he may answer that he is blind. When requested to put out his tongue, he is seen to make an effort to do so. Sometimes the only response is a movement of the lips; at other times the tip of the tongue is protruded, which is then seen to be dry, and covered with a whitish fur, through which some few red papillæ are seen to project; but it is not deviated to the side. When food is put into his mouth, the patient makes an effort at deglutition, and generally succeeds in swallowing small quantities of fluid. The eyes are closed. On opening the lids, the eye-balls are seen to be deeply retracted into the orbit, one sometimes more so than the other; and they are seen to diverge somewhat in their direction, which imparts to them a particularly dazed and stupid expression. The deeper the coma, the greater is, *cæteris paribus*, the degree of divergence. The pupils are small and insensible to light. On account of the position of the eyes, an ophthalmoscopic examination is generally not practicable, but reveals nothing unusual when practiced. The reflex excitability of the conjunctivæ is either very much blunted or entirely gone. The breath is sometimes offensive.

"The muscles of the limbs and the body are in a state of perfect relaxation. The body will retain any position which is given it. On lifting the arms or legs, no resistance is encountered; and on dropping them, they flop back heavily by their own weight, like inanimate matter, as in a dead body from which rigor has disappeared. There is no difference at all between the two sides of the body; no appearance of hemiplegia, or rigidity, or tremor, but a dead level of paralysis, with complete loss of muscular tone, everywhere.

" Sensibility and reflex excitability are greatly diminished, or quite gone. I have already mentioned that the conjunctival reflex is lessened or absent, and that there is no light reflex in the pupils. Tickling the soles or the knees produces no withdrawal of the legs; but on smartly pricking the skin with a pin, there is generally a slight response. Where the coma is not very deep, the patient may express, by a grunt, his dislike of the proceeding. The deep reflexes or tendon phenomena are either absent, or can only be elicited with considerable difficulty, and then appear slight and sluggish.

" There is, either from the first or very soon, incontinence of the excretions, especially of the urine, which is apparently secreted much in the usual manner, and dribbles away as it reaches the bladder, through paralysis of the sphincter. The fæces are also apt to come away involuntarily, but occasionally there is obstinate constipation, which only yields to powerful purgatives or enemata, and the evacuation then takes place into bed, the patient having no sensation of its coming away, and being unable to give a warning.

" The pulse is habitually slow, beating at the rate of forty, fifty, or sixty in the minute. In one case I have known it to go down to thirty-six, while in another it was eighty-six. The quality of the pulse varies in the different cases; it may be hard and wiry, showing the sphygmographic signs of increased tension, or tolerably full, or small and feeble, when the sphygmograph indicates low tension.

" Respiration is slow and shallow, the excursion of the chest-walls and diaphragm being insignificant. The rate of inspirations varies like that of pulsation, but is generally less than in health. The average rate appears to be from eight to ten.

" The temperature is below the average, and ranges habitually between 96° and 97°. In one case I have known it to go down to 95°.

"In two cases there was an eruption of herpes in the face, large groups of vesicles being formed on inflamed patches on both cheeks. On the first day the liquid was clear; on the second it became opaque, and the epidermis then gradually peeled off in small patches. Otherwise the skin is generally dry, there being little or no perceptible perspiration.

"What is the condition of the brain in the cases which I have just described? It is evidently a complex one, for while we have, on the one hand, symptoms of paralysis, there are, on the other hand, signs of irritation of the nervous centres. The loss of consciousness, and of voluntary motion and sensation, shows that the function of the cineritious substance of the hemispheres, and notably that of the frontal and temporal lobes and of the central convulsions, is in abeyance; while the state of the pulse, the respiration, and temperature shows that the cardiac, vaso-motor, respiratory, and thermic centres in the medulla oblongata and the pons Varolii are in a state of irritation. Such a coincidence of paralysis and irritation is by no means so singular as it might appear at first sight, if we consider the various degrees of excitability which exist normally in different portions of such a highly complex organ as the brain is known to be. Of all parts of the encephalon, the gray cortex is the most highly vitalized, and the one that requires the most active circulation of the blood, the most incessant supply of oxygen, in order to be able to properly discharge its function. Any interference with the supply of arterial blood to the cortex, however temporary, acts like a blow with a hammer on a magnet, that is to say, it destroys its function for the time being by suddenly disturbing its molecular condition. In poisoning by carbonic acid, there is at first a short stage of irritation of the cortex, shown by headache, giddiness, and noises in the head; but this is rapidly succeeded by depression, consciousness being lost and a state of coma induced. At this time, however, there is still irritation of the medulla, shown by a slow pulse, increased blood-pressure, and convulsions, and this stage is only eventually succeeded by paralysis, when respiration becomes feeble, the blood-pressure falls, and death results from apnœa. That in patients suffering from syphilitic coma there may be a short stage of cortical irritation, is rendered probable by the premonitory symptoms which I have mentioned, such as headache, giddiness, and a feeling of confusion.

"A coincidence of depression and irritation may be observed in much less complex structures than the brain. Thus we have, in the early stage of an acute attack of sciatica—whether of rheumatic or traumatic origin—on the one hand, symptoms of depression, viz., numbness in the foot and loss of power in the muscles supplied by the sciatic nerve; and, on the other hand, concurrently with them, symptoms of irritation, viz., acute pain in the whole or part of the limb, and convulsive twitches in the muscles which are under the influence of the suffering nerve.

"That there is irritation of the lower centres in the first stage of syphilitic coma seems to me to be proved by the slow pulse, the increase of blood-pressure which is present in the majority of cases, the retarded respiration, the lowered temperature, and finally by the state of the pupil and of the ocular muscles. There is a centre for these latter parts in the posterior portion of the floor of the third ventricle and the aqueduct of Sylvius, and, therefore, an intimate connection with the upper portion of the pons Varolii, which has by Hensen and Voelcker been shown to have definite relations to the iris and the other muscles of the eye. Irritation of this centre would explain the contraction of the pupil and the different forms of ocular spasm which are found to be present in syphilitic coma; just as paralysis of the same centre is known to lead to the different forms of ophthalmoplegia.

"The initial stage of syphilitic coma lasts in general from two to five days, and is followed either by recovery, or merges into the final stage which leads to death. In the former case the patient gradually begins to show signs of returning consciousness; he opens his eyes from time to time, moves them about in different directions, and recognizes the people about him. He regains his command over the sphincters, and calls the nurse when desiring to pass his excretions. The power of swallowing is improved; he begins to take food with some amount of relish, recovers his muscular power, sits up in bed, has natural, refreshing sleep followed by wakefulness, and presently wants to get up, and begins to go about again. In ten days or a fortnight he may apparently be well, and able to resume, at least to some extent, his previous occupations.

"In other cases recovery is more slow and imperfect. The speech remains indistinct and halting, the memory is week. An hour after having seen a person or read a newspaper, the patient remembers nothing about it. The power of moving the eyes freely remains impaired. He does not seem to take much interest in his affairs, or show much affection for his family. At times, however, he appears to realize his position acutely, and bursts out crying, while at other times he is absent-minded and drowsy.

> 'All is but toys; renown and grace is dead;
> The wine of life is drawn, and the mere lees
> Is left this vault to brag of.'

Eventually, however, even in these less favorable cases the brain power may be more or less restored, showing but little deterioration compared with what it was previous to the attack.

"This is the bright side of the picture; and, as recovery generally takes place in consequence of an energetic specific treatment, the doctor may well take credit to himself for having saved his patient's life or reason. But there is also a darker side, since in some cases all the resources of our art prove unavailing. The patient then, after having been for a few days in the condition previously described, gradually sinks into what I propose to call the *final stage* of syphilitic coma.

"3. This stage is characterized by an intensification of the symptoms of unconsciousness, loss of voluntary power, sensation, and reflex excitability; while the signs which I have referred to irritation of the pons and bulb now pass into such as denote a paralytic state of these organs. The face is livid and cyanosed; the conjunctivæ are injected, covered with shreds of mucus, and insensible to touch or irritation. The mouth is wide open, from paralysis of the masseter muscles, causing the lower jaw to drop. The breath is fetid; the power of swallowing lost. There is either excessive secretion of buccal mucus, or great dryness of the lips, tongue, and cavity of the mouth, which are often covered with sordes. The surface of the body is bathed in clammy sweat. The pulse, where it has been hard and wiry, rapidly loses that character, and becomes small, feeble and very quick, going up to 140, 180 and more. It eventually cannot be counted, and shows sphygmographically the characters of collapse, there being only a very slight elevation followed by a proportionate depression, but without waves, aortic notch or dicrotism. Respiration from having been retarded, is now accelerated, with thirty to forty and more inspirations per minute. It may become stertorous, and pass into Cheyne-Stokes type; or neurolytic catarrh of the air-passages sets in, with excessive secretion of bronchial, tracheal, and laryngeal mucus, while on auscultation râles are heard all over the chest. This may pass into hypostatic pneumonia, when the mucus becomes tinged with blood. At the same time, the temperature is found to rise from 95° and 96° to 104°, 106° and 108°. The pupils become enlarged, and show their maximal dilatation at the moment of death. Eventually, the face assumes the Hippocratic expression, and is occasionally so altered within a few minutes that the patient's friends have a difficulty in recognizing him. This stage generally lasts from twenty-four to thirty-six hours, and terminates in dissolution; the patient passes away to

> 'The undiscovered country from whose bourne
> No traveler returns.'

"Of the eight cases of syphilitic coma which have fallen under my observation, six ended in recovery, and two in death, in the first attack. In three of those who recovered from the first attack, however, relapses took place after some time, and one of these latter patients eventually died, after having survived five such attacks in three years."

As to prognosis, we were formerly led to believe that this disease, in any form, was always fatal. It was contended later that only one-third recovered, while now, from experience and the literature on the subject, we conclude that it is curable in over sixty per cent. of the cases treated. As to treatment, we must keep in mind, always, the impoverished state of the brain and the probability of disturbances of digestion. With these in view, we are ready to administer anti syphilitic treatment. Chief among the drugs is iodide of potash, in large doses, commencing at thirty grains, increasing rapidly until we get iodism. To do this, we may need to give as large as fifty to sixty grain doses, three times daily. If disturbances of the stomach arise, we may administer the drug partly before meals and partly after. The combination with carbonate of ammonia is a favorite way of administration; with this it is said to increase the effect of the drug three-fold, the proportion of the ammonia being about one to three in ordinary doses, but, of course, it cannot and need not be increased like the potassium iodide. Mercury in various forms, preferable by inunction, should be vigorously employed from time to time.

